# Porcine reproductive and respiratory syndrome virus infection induces endoplasmic reticulum stress, facilitates virus replication, and contributes to autophagy and apoptosis

**DOI:** 10.1038/s41598-020-69959-z

**Published:** 2020-08-04

**Authors:** Quangang Chen, Yanjuan Men, Dang Wang, Deqin Xu, Suyan Liu, Shaobo Xiao, Liurong Fang

**Affiliations:** 1 School of Life Science, Xuzhou Medical University, Xuzhou, 221004 China; 2 State Key Laboratory of Agricultural Microbiology, College of Veterinary Medicine, Huazhong Agricultural University, Wuhan, 430070 China; 3 Key Laboratory of Preventive Veterinary Medicine in Hubei Province, The Cooperative Innovation Center for Sustainable Pig Production, Wuhan, 430070 China; 4 Department of Oncology, The Affiliated Hospital of Xuzhou Medical University, Xuzhou, 221004 China

**Keywords:** Zoology, Virology

## Abstract

During viral infection, the host cell synthesizes high amounts of viral proteins, which often causes stress to the endoplasmic reticulum (ER). To manage abnormal ER stress, mammalian cells trigger a response called the unfolded protein response (UPR). Previous studies have indicated that porcine reproductive and respiratory syndrome virus (PRRSV), an Arterivirus that has been devastating the swine industry worldwide, can induce ER stress and activate UPR, however, the activation pathways and the biological significance requires further investigation. In this study, we demonstrated that, among the three types of UPR pathways, PRRSV infection induced PERK and IRE1 pathways, but not the ATF6 pathway. Furthermore, the induction of UPR promoted PRRSV replication. We also found that PRRSV-induced UPR, particularly the PERK pathway, was involved in the induction of autophagy, a cellular degradation process that can alleviate cell stress. Besides, we also provided insights into the ER stress-mediated apoptosis in response to PRRSV infection. PRRSV infection induced the expression of the transcription factor CHOP, which activated caspase 3 and PARP led to ER stress-mediated apoptosis. Using 3-Methyladenine (3-MA) to inhibit autophagy, the increased ER stress and cell apoptosis were observed in the PRRSV infected cell. Taken together, our results revealed the associations of ER stress, autophagy, and apoptosis during PRRSV infection, helping us to further understand how PRRSV interacts with host cells.

## Introduction

Porcine reproductive and respiratory syndrome (PRRS) caused by PRRS virus (PRRSV), is one of the most economically significant disease in the swine industry^1^. PRRSV belongs to the Nidovirales order, Arteriviridae family of positive-sense single-stranded RNA viruses^2^. The burden of PRRSV infection on the host cell has been shown to initiate a number of cellular stress responses. Here, we focused on the endoplasmic reticulum (ER) stress during PRRSV infection.

The ER is an extensive membranous network that provides a unique environment for the synthesis, maturation, and proper folding of a wide range of proteins. It also plays a critical role in the regulation of calcium concentration and intracellular signal transduction. Endogenous imbalances in cells, such as the accumulation of misfolded or unfolded proteins, can cause a stress to the ER system. To alleviate this stress, the unfolded protein response (UPR) is activated. The UPR eliminates misfolded or unfolded proteins in different ways: (1) upregulating the expression of chaperone proteins to enhance the folding capability or (2) inducing the expression of degradation factors to enhance the endoplasmic reticulum associated protein degradation (ERAD). Additionally, the UPR can inhibit protein translation to help the ER to cope with the stress. In mammals, this signal transduction cascade is mediated by three types of ER transmembrane proteins: protein kinase RNA (PKR)-like ER kinase (PERK), activating transcription factor-6 (ATF6), and inositol-requiring enzyme 1 (IRE1).

PERK is an ER-localized type I transmembrane protein; it is maintained in an inactive monomeric state by binding to GRP78. Once activated by ER stress, PERK dissociates from GRP78 and phosphorylates itself; active PERK leads to phosphorylation of the translation initiation factor eIF2α^3,4^. In its phosphorylated form, eIF2α decreases global translation by tightly binding to another initiation factor^5^. Interestingly, the phosphorylation of eIF2α can activate the activating transcription factor-4 (ATF4), thus leading to the upregulation of GADD34, whose activity dephosphorylates eIF2α, thus relieving translation attenuation and promoting protein synthesis^6,7^.

Similarly, in response to ER stress, IRE1 dissociates from GRP78, leading to its autophosphorylation and activation. Active IRE1 splices out a 26-nucleotide intron from XBP1 mRNA; this splicing creates a translational frameshift and generates a spliced variant XBP1s^8,9^. XBP1s is an active transcription factor that can induce the expression of a subset of genes encoding chaperones and degradation enzymes by binding to ER stress elements (ERSE) or UPR elements (UPRE) (e.g., EDEM)^10^.

ATF6 is a type II transmembrane protein; ER stress causes the inactive ATF6 translocates to the Golgi, and cleaved by proteases into the active form. The cleaved ATF6 translocates to the nucleus and binds to the ERSE in genes encoding ER chaperone proteins such as GRP78 and GRP94. This binding can enhance the expression of these proteins and hence increase protein folding activity in the ER^11^. In addition, ATF6 regulates other important targets, including XBP1, as well as many ER chaperone-encoding genes^8^.

The UPR is a prosurvival signaling pathway to restore ER homeostasis, and cells under severe ER stress are able to recruit survival pathways such as autophagy, which is a catabolic process involving degradation of long-lived macromolecules and defective organelles. UPR-induced autophagy has been thoroughly described in various systems. On the other hand, if the overload of unfolded or misfolded proteins in the ER is not resolved, the excessive level of UPR will lead to cell apoptosis. The activation of the c/EBP homologous protein (CHOP) is the hallmark of ER stress-mediated apoptosis. Under ER stress, both PERK and ATF6 pathways can activate the expression of CHOP.

Previous studies have shown that PRRSV infection induced UPR^12,13^, however, the approach used by PRRSV to induce UPR, and the biological significance of PRRSV-induced UPR need further investigations. In the present study, we showed that PRRSV infection activated PERK and IRE1 pathways, but not the ATF6 pathway. Moreover, our results provide insights into the ER stress-mediated apoptosis in response to PRRSV infection and indicate that CHOP plays a key role in ER stress-mediated apoptosis. In addition, several reports have indicated that PRRSV infection can trigger autophagy, and in the present study, we found that PRRSV-induced autophagy was most probably indirectly activated through ER stress, and contributed to alleviate the stress status.

## Results

### PRRSV infection induces ER dilation and upregulates GRP78 and GRP94 expression

**Figure 1:**
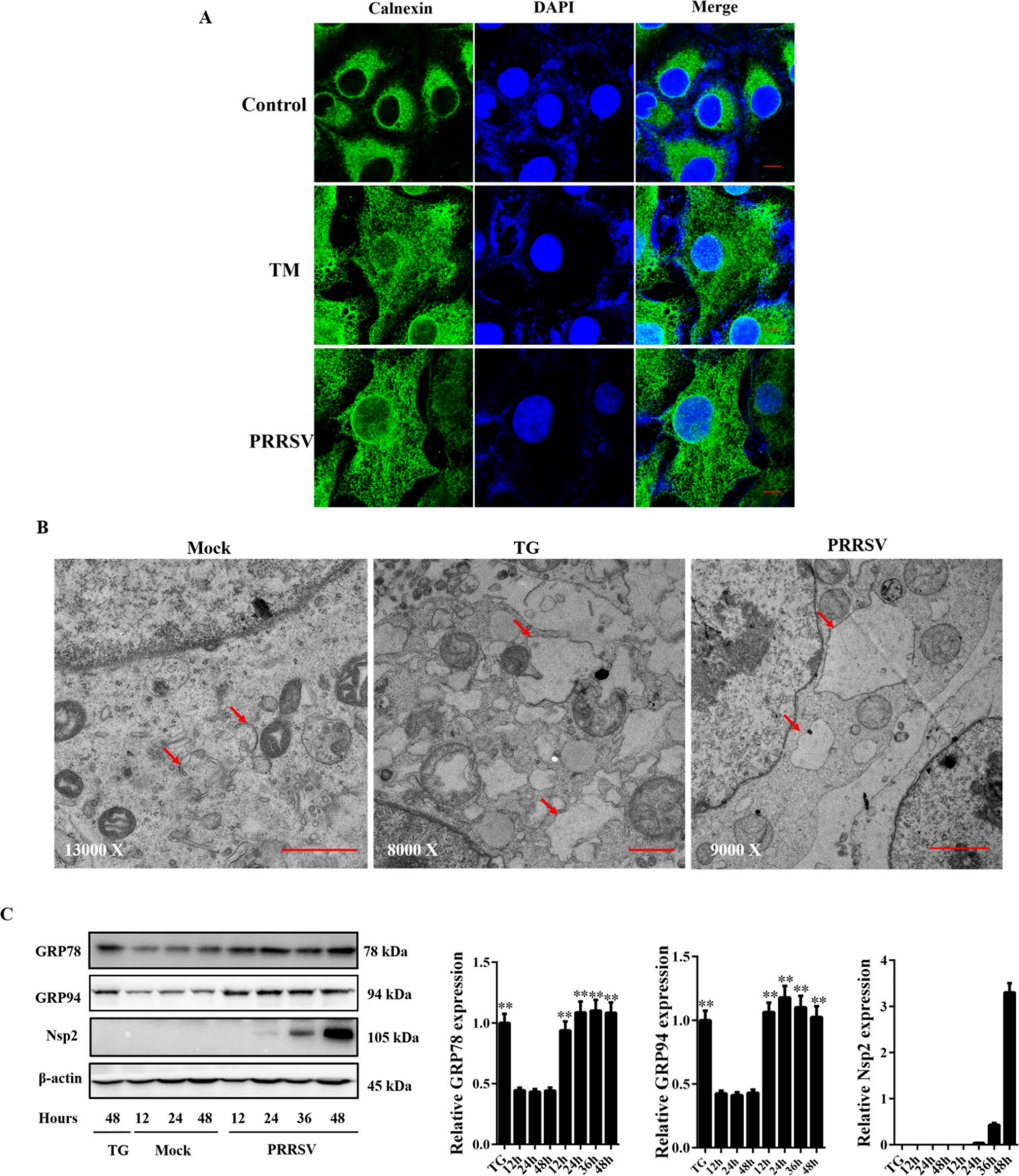
PRRSV infection can induce ER stress. Marc-145 cells were mock-infected or infected with PRRSV for 36 h at an MOI of 0.5, and cells treated with TG (5 μM) were used as the positive controls. (**A**) Immunofluorescence assay was performed to observe the ER morphology. Scale bars, 10 μm. (**B**) Cells were observed by electron microscopy observation. The ER (arrows) in the cells are indicated. Scale bars, 1 μm. (**C**) Cells were collected for western blot analysis at the indicated time points, using monoclonal antibodies against Nsp2, GRP78 and GRP94. β-actin expression was used as the protein loading control. The bar graph represents the quantitative analysis of western blots. The amount of protein was normalized to β-actin. Error bars indicate means ± SD from three independent experiments. **p < 0.01.

Several studies have reported that cells expand their ER volume under UPR-inducing conditions^14^. To visualize the ER of cells during PRRSV infection, we investigated ER morphology by immunofluorescence microscopy using an antibody against the ER marker protein, calnexin. We found that TG treatment or PRRSV infection caused massive ER expansion (Fig. 1A). To observe cellular changes that may accompany the response to PRRSV infection, ultrastructural analysis by electron microscopy was performed. As shown in Fig. 1B, similar to the positive control, the most prominent change observed in the PRRSV-infected cells was the expansion of the ER. These results suggested that ER stress would be induced during PRRSV infection. As the hallmark of ER stress, cells usually activate the expression of ER chaperones to increase the folding capacity of the ER^15^. To study the effects of PRRSV infection on ER homeostasis, we detected the expression of GRP78 and GRP94 at various time points post-infection (pi). As shown in Fig. 1C, the expression of both GRP78 and GRP94 was enhanced at various time points with the progression of PRRSV infection, which was verified by detecting the expression of PRRSV Nsp2. Taken together, the results indicated that PRRSV infection induced ER stress.

### PRRSV infection activates PERK signaling

**Figure 2:**
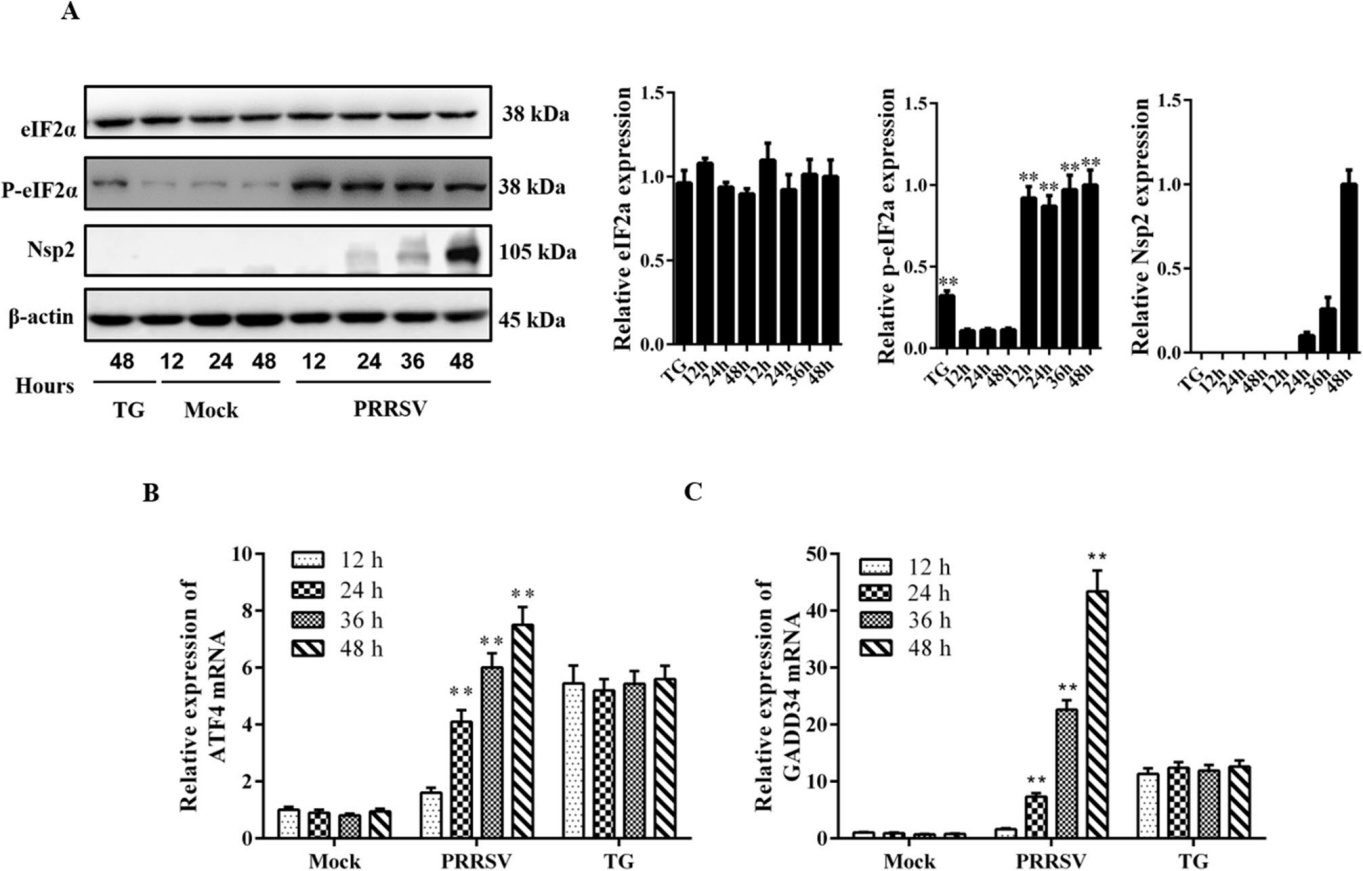
PRRSV infection activates the PERK signaling branch. Marc-145 cells were mock-infected or infected with PRRSV at an MOI of 0.5, and then harvested at the indicated time points. Cells treated with TG (5 μM) for 4 h, and collected at the indicated time points as the positive control. (**A**) Cell lysates were evaluated by western blot using antibodies specific for the phosphorylated forms of eIF2α, total eIF2α, Nsp2, and β-actin (loading control). The relative expression of p-eIF2α was analyzed with ImageJ based on the band intensities. Error bars indicate mean ± SD from three independent experiments. (**B**,**C**) RNA was extracted from cells at the indicated time points, and analyzed by quantitative RT-PCR using primers specific for ATF4 and GADD34. The fold changes of ATF4 and GADD34 were relative to mock-infected cells (set at 1). **p < 0.01.

Chen et al. have demonstrated that PRRSV infection can induce the phosphorylation of PERK, which favors the phosphorylation of eIF2α. To investigate whether the PERK signaling pathway is activated during PRRSV infection, we further tested the downstream gene expression during PRRSV infection. Marc-145 cells were infected with PRRSV and harvested at the indicated time points. Cell lysates were analyzed by western blot assays. Similar to the previous studies the expression of the phosphorylated form of eIF2α was enhanced at 12 hpi and remained elevated throughout the course of infection (Fig. 2A). In addition, total eIF2α was determined by an antibody that recognizes both phosphorylated and unphosphorylated eIF2α, and we found that PRRSV infection did not affect the levels of total eIF2α. Next, we investigated whether a limited amount of phosphorylated eIF2α in PRRSV-infected cells was sufficient to induce translation of ATF4. The qRT-PCR was performed to measure the levels of ATF4 and GADD34 mRNA, which can be induced by eIF2α. The results showed a high increase in the level of ATF4 and GADD34 mRNA after PRRSV infection or treatment with TG (Fig. 2B,C). Taken together, the results implied that PRRSV infection activated the PERK signaling pathway, which may result in a translational attenuation.

### PRRSV infection activates the IRE1/Xbp1 branch of UPR

**Figure 3:**
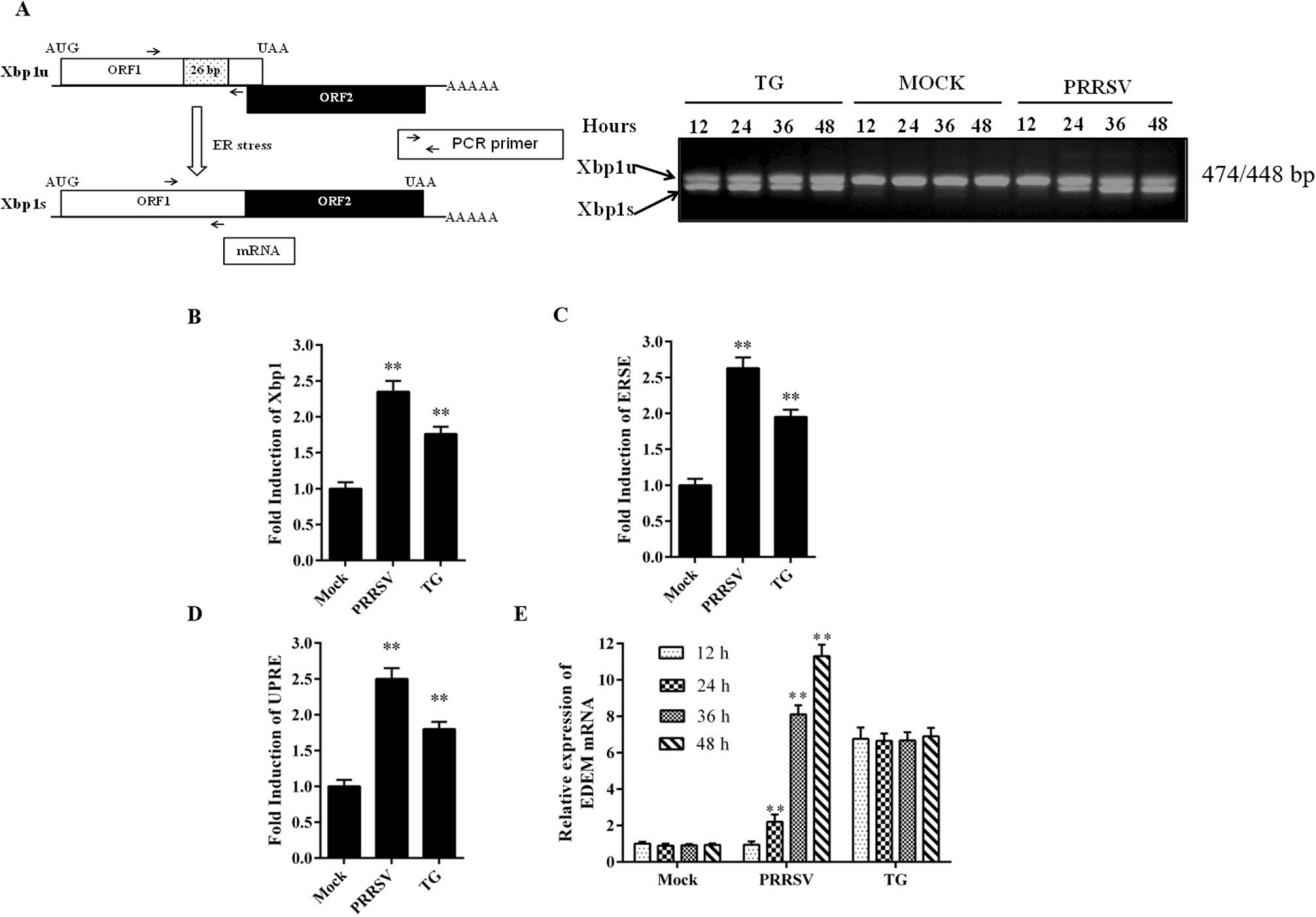
The IRE-1 signaling branch was induced during PRRSV infection. (**A**) PRRSV infected Marc-145 cells were harvested at the indicated time points, and total RNAs were extracted. Both the unspliced and spliced forms of Xbp-1 were analyzed by RT-PCR. Mock-infected and TG-treated (5 μM) Marc-145 cells were used as negative and positive controls respectively. PCR products were separated by electrophoresis on a 3% agarose gel and visualized by ethidium bromide staining. (**B**–**D**) Marc-145 cells were seeded in the 24 well plates, and transfected with 0.1 μg/well of the reporter plasmid UPRE-luc, ERSE-luc or Xbp1-luc, along with 0.05 μg/well of pRL-TK plasmid, followed by PRRSV infection. Thirty-six hours after infection, the cells were collected and luciferase activities were determined. (**E**) Marc-145 cells were mock-infected or infected with PRRSV at an MOI of 0.5, and cells were collected at the indicated time points, the mRNA expression level of EDEM was evaluated by qRT-PCR. Cells treated with TG (5 μM) for 4 h were used as the positive control. Results are expressed as mean ± SD for three independent experiments with three technical replicates per group. **p < 0.01.

It has been reported that in response to ER stress, active IRE1 splices a 26-bp intron from Xbp1u, resulting in an active Xbp1s, which acts as a potent transcriptional activator of several genes involved in the UPR. To determine whether PRRSV infection can trigger the IRE1 pathway, we tested the splicing of the Xbp1 mRNA by RT-PCR using primers overlapping the 26-bp intron. As shown in Fig. 3A, the 448-bp fragment amplified from Xbp1s mRNA was detected starting at 24 h after PRRSV infection, and the level of this mRNA continued to increase through 48 h. As expected, a similar band was also observed in TG-treated cells but not in mock-infected cells. The 474-bp fragment corresponding to Xbp1u was also generated from total mRNA prepared from all the cells including the control (Fig. 3A). Furthermore, the dual-luciferase reporter gene was used to evaluate the cleavage level of Xbp1. Under normal conditions, the Xbp1-luc mRNA remains unspliced, and the translation was terminated at the stop codon located upstream of luciferase mRNA. Under ER stress conditions, the Xbp1-luc transcript was spliced, leading to a frameshift, and resulting in full Xbp1-luc-fused protein expression. Subsequently, Xbp1-luc and pRL-TK were cotransfected into Marc-145 cells, followed by mock infection or infection with PRRSV at an MOI of 0.5. Cells treated with TG for 4 h were used as a positive control. As shown in Fig. 3B, luciferase activity was elevated approximately twofold in cells treated with TG or infected with PRRSV. This further demonstrated that PRRSV infection induced the splicing of Xbp1 mRNA.

Because the spliced form of Xbp1 encodes a transcription factor, we investigated whether the downstream target genes were transcriptionally activated in PRRSV-infected cells. First, we detected the activation of ERSE and UPRE, and the results indicated that both the response elements were activated significantly during PRRSV infection (Fig. 3C,D); a similar finding was noted for TG-treated cells. Hence, we suspected that the Xbp1 target genes would be induced by PRRSV. This hypothesis was confirmed by elevated mRNA expression of EDEM after PRRSV infection (Fig. 3E). These results indicated that PRRSV infection activated the IRE1 pathway, leading to the splicing of Xbp1 RNA and resulting in the activation of downstream target genes.

### PRRSV infection cannot activate the ATF6 pathway

**Figure 4:**
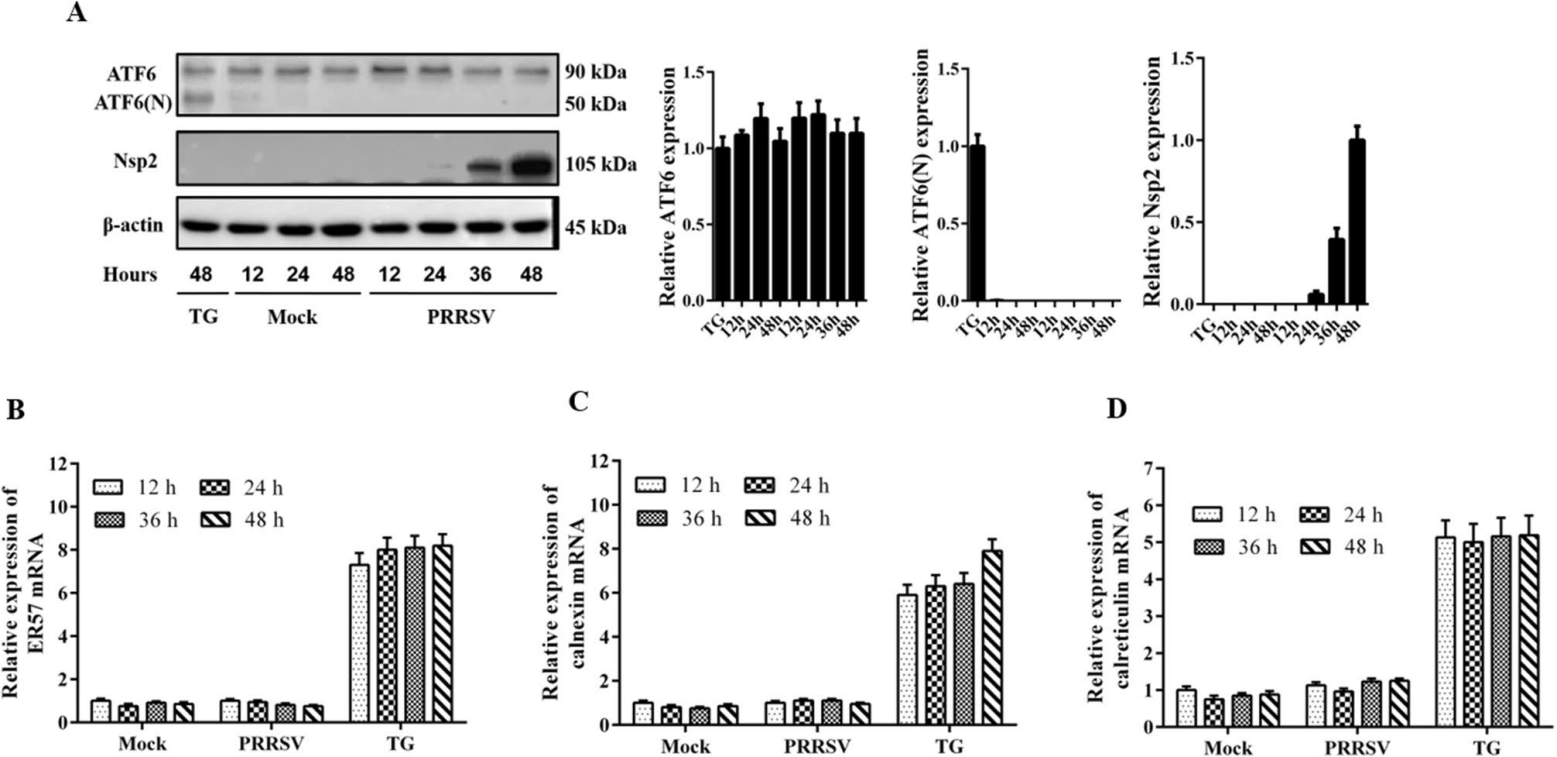
PRRSV infection cannot activate the ATF6 signaling pathway. (**A**) Marc-145 cells were seeded, and mock-infected or infected with PRRSV. Next, the cells were collected and equal amounts of cellular lysates were subjected to SDS-PAGE and immunoblotted with anti-ATF6 and anti-Nsp2 antibodies. For the positive control cells were treated with TG (5 μM) for 4 h, and collected at the indicated time points. β-actin expression was used as a protein loading control. The relative expression of ATF6(N) was analyzed with ImageJ based on the band intensities. The TG-treated group was set at 1. Error bars indicate mean ± SD from three independent experiments. (**B**–**D**) Total cellular RNA was extracted from the cell using the TRIzol reagent and subject to ER57, calnexin, and calreticulin-specific qRT-PCR. Cells treated with TG (5 μM) for 4 h were used as the positive control. **p < 0.01.

In response to ER stress, a 90-kDa precursor of ATF6 is cleaved into a 50-kDa protein that functions as a transcription factor. To examine the effects of PRRSV infection on the ATF6 pathway, a western blot assay was performed to assess the status of ATF6. As previously reported, by using an antibody that recognizes the N terminus of ATF6 (recognizes both 90-kDa and 50-kDa protein), both 90- and 50-kDa ATF6 proteins were detected in Marc-145 cells treated with TG for 4 h. However, in mock-infected cells, the 50-kDa cleavage products were undetectable at any time during PRRSV infection (Fig. 4A). These results suggested that PRRSV infection did not induce the cleavage of ATF6.

On the basis of this result, we predicted that the ATF6 target genes would not be induced in PRRSV-infected cells. To confirm this prediction, qRT-PCR was performed to evaluate the mRNA expression levels of molecular chaperones regulated by ATF6. As expected, treatment with TG induced the expression of ER57, calnexin, and calreticulin. However, there was no obvious change in the expression levels of these genes during PRRSV infection (Fig. 4B–D). These results demonstrated that PRRSV infection induced ER stress but could not activate the ATF6 pathway.

### PRRSV-induced ER stress modulates its replication

**Figure 5:**
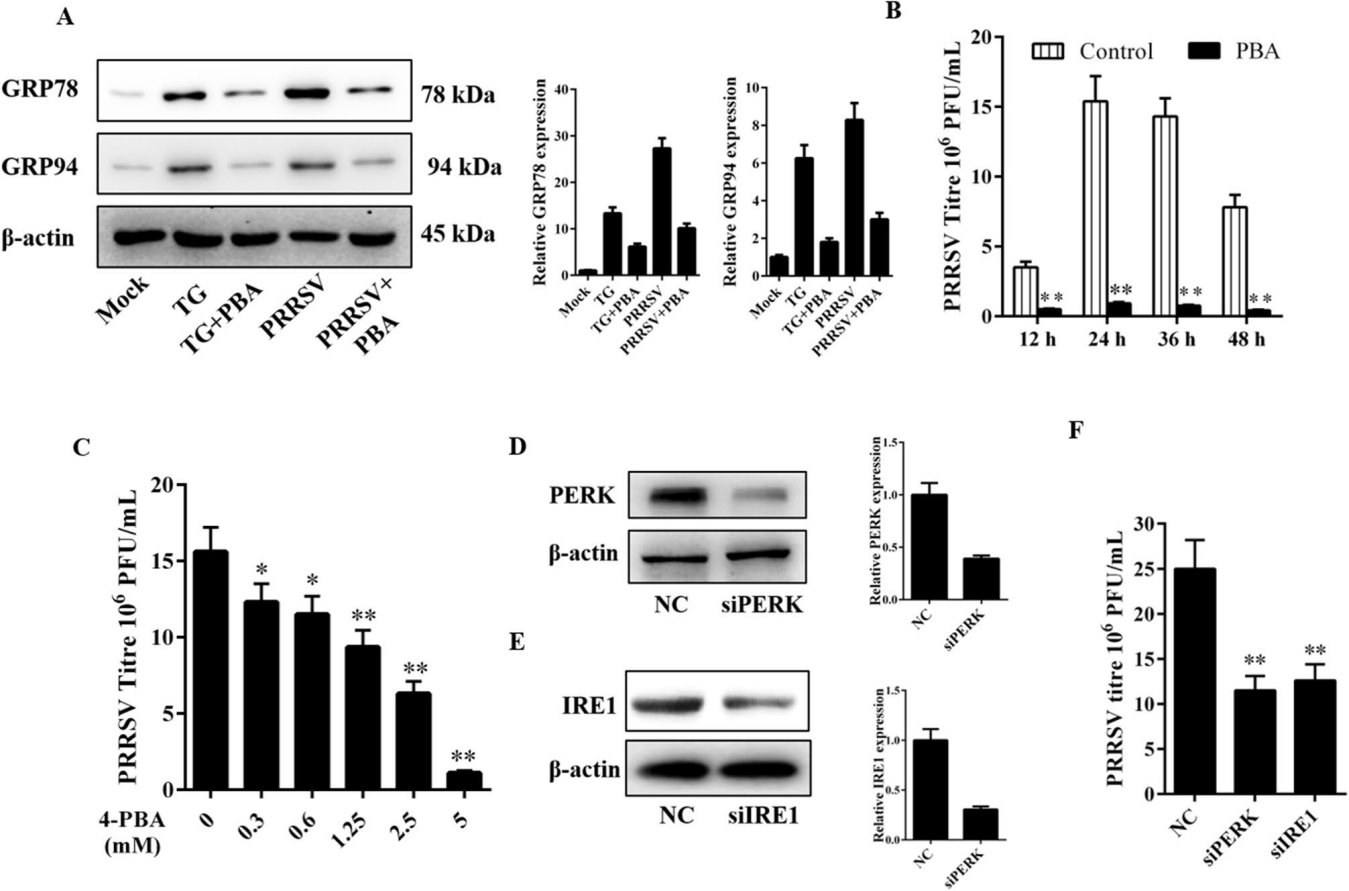
UPR induction benefits PRRSV replication. (**A**) Marc-145 cells were treated with 4-PBA (5 mM), and infected with PRRSV for 36 h. GPR78 and GRP94 protein expression was evaluated by western blot. The relative expression was analyzed with ImageJ based on the band intensities. (**B**) Marc-145 cells were treated with 4-PBA, and infected with PRRSV. After that, cells were collected at the indicated time points, and the viral titers were determined by plaque assay. (**C**) Cells were pretreated with 4-PBA at different concentrations for 1 h, and then infected with PRRSV, 36 h later, virus proliferation was determined by plaque assay. (**D**,**E**) Marc-145 cells were transfected with the indicated siRNA. After 24 h, western blot was performed to analyze the expression of PERK and IRE-1. The relative expression of PERK and IRE1 was analyzed with ImageJ based on the band intensities. (**F**) Marc-145 cells were transfected with siRNAs targeting PERK or IRE-1 for 24 h, followed by PRRSV infection, Thirty-six hours post-infection, the cells were collected to determine viral replication by plaque assay. Results are expressed as mean ± SD for three independent experiments with three technical replicates per group. *p < 0.05 and **p < 0.01.

We demonstrated that upon PRRSV infection, both PERK and IRE1 branches of the UPR were activated as a host response. Next, we determined whether the modulation of the UPR could have an effect on PRRSV replication. 4-PBA, a small bioavailable molecule, referred to as a small molecular chaperone that can decrease ER stress response signaling was used in this experiment. First, we verified whether 4-PBA can alleviate PRRSV-induced ER stress. We found that 4-PBA treatment can significantly inhibit GRP78 and GRP94 expression induced by TG treatment or PRRSV infection (Fig. 5A), indicating the ER stress inhibiting effects of 4-PBA in MARC-145 cells. Then, we treated Marc-145 cells with 4-PBA for 1 h prior to infection with PRRSV, and the cells were collected at different time points (12, 24, 36, and 48 hpi) after PRRSV infection. Similarly, other cell samples treated with different doses of 4-PBA were collected at 24 hpi. Virus titers were then determined by plaque assay. As shown in Fig. 5B,C, regardless of the time- or dose-dependent effect of 4-PBA, the data showed that 4-PBA reduced PRRSV growth significantly at micromolar concentrations.

To extend these studies, we performed RNA interference (RNAi) to knockdown PERK and IRE1 and evaluated its effect on PRRSV replication. First, western blot was performed to evaluate the efficiency of siRNA, compared with control siRNA, the siRNA targeting PERK and IRE1 significantly suppressed the expression of the corresponding target (Fig. 5D,E). The propagation of PRRSV was evaluated after knockdown of PERK or IRE1, and the results suggested that knockdown of PERK or IRE1 significantly decreases PRRSV yield (Fig. 5F). On the basis of these results, we concluded that the UPR induced by PRRSV played a positive role in its replication.

### ER stress is involved in PRRSV-induced autophagy

**Figure 6:**
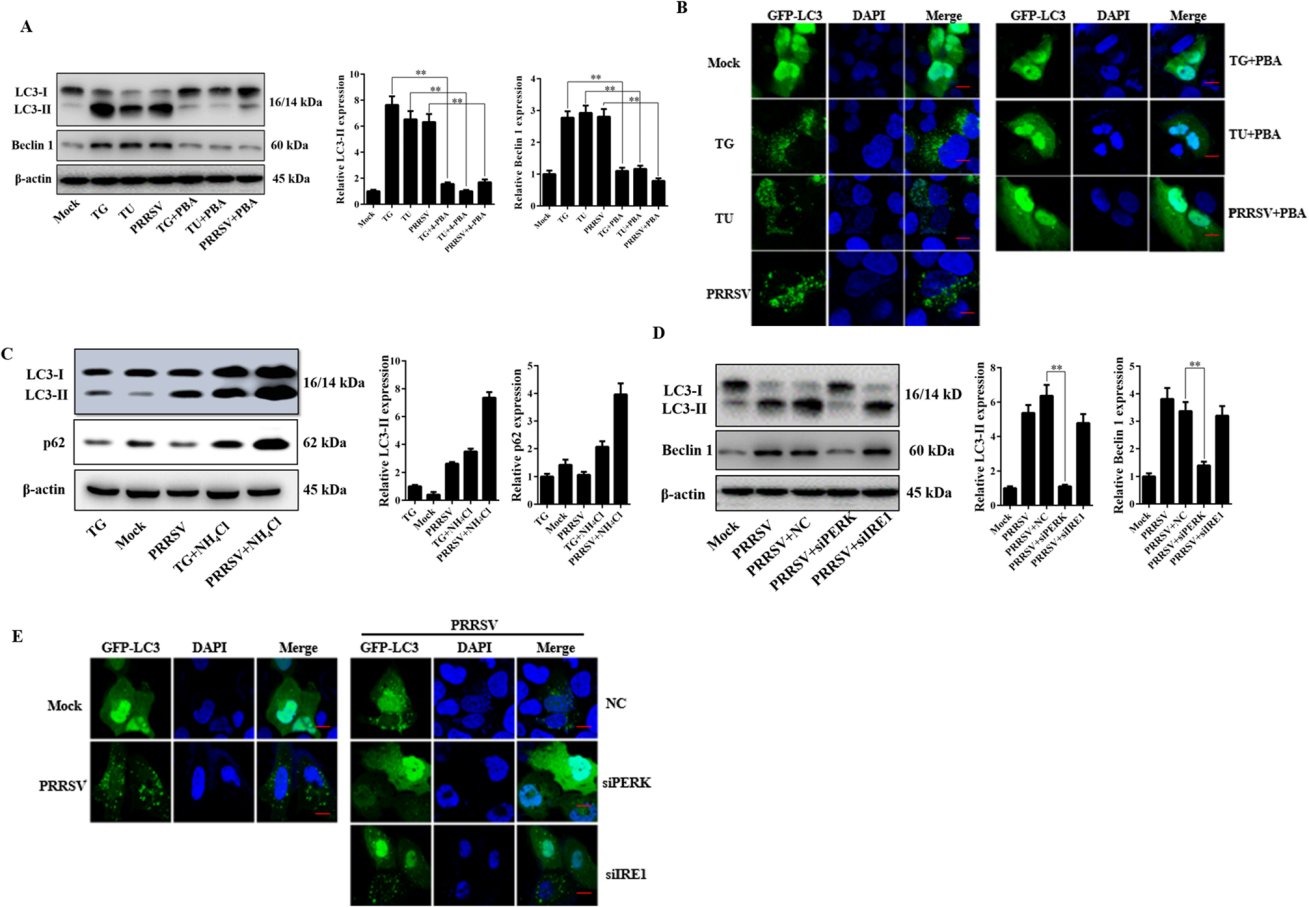
Autophagy was induced during PRRSV infection through ER stress. (**A**) Marc-145 cells were seeded onto plates and treated as labeled. Cell lysates were then prepared for the analysis of the LC3-II and beclin 1 expression. The graph shows the quantitative analysis of the relative expression of LC3-II and beclin 1 to β-actin respectively. (**B**) Marc-145 cells were transfected with the GFP-LC3 plasmid. After 24 h, cells were infected with PRRSV and treated as labeled. Thirty-six hours post-infection cells were observed by fluorescence microscopy. Scale bars, 50 μm. (**C**) Marc-145 cells were infected with PRRSV and treated by NH_4_Cl (10 mM) for 36 h, the LC3 and p62 expression was evaluated to access the autophagic flux. The relative protein level was analyzed and shown in the right panel. (**D**) Marc-145 cells were transfected with siRNAs targeting PERK or IRE-1 for 24 h, followed by PRRSV infection. At 36 h post-infection, cell lysates were collected, the expression of LC3-II and beclin 1 was evaluated by western blot. The right panel shows the statistics for the relative LC3-II protein level. (**E**) Marc-145 cells were cotransfected with GFP-LC3 and siRNAs, 24 h after transfection, PRRSV was inoculated, and the cells were observed by fluorescence microscopy at 36 h post-inoculation. Scal bars, 50 μm. Error bars indicate mean ± SD from three independent experiments. **p < 0.01.

From the above results, we determined that PRRSV infection can induce ER stress and activate UPR. Recently, several research groups, including our group, have revealed that PRRSV infection can activate autophagy. To elucidate whether autophagy induction was related to ER stress, a western blot assay was performed to detect the expression of beclin 1 and conversion level of LC3-I to LC3-II. As shown in Fig. 6A, an increase in the amount of LC3-II and beclin 1 was observed in cells treated with TG (5 μM) and TU (5 μg/mL) or infected with PRRSV; however, when cells were treated with 4-PBA to decrease ER stress response, the conversion of LC3-I to LC3-II and the expression of beclin 1 was highly inhibited. In addition, punctate accumulation of LC3, which indicates the recruitment of LC3-II to autophagic vacuoles, was also detected (Fig. 6B). All these results indicated that PRRSV-induced ER stress contributed to the accumulation of the autophagosome. The accumulation of autophagosomes could indicate either autophagic activation or a blockage of downstream steps in autophagy, such as inefficient fusion or decreased lysosomal degradation. Hence, we then monitor the LC3-II and p62 expression to measure the autophagic flux during PRRSV infection, as shown in Fig. 6C, the results indicated that PRRSV infection can promote autophagic flux. After establishing that autophagy occurred via ER stress, we further verified which branch of UPR was related to the induction of autophagy. We previously demonstrated that PRRSV infection activated PERK and IRE-I signaling but not the ATF6 pathway. Hence, we knocked down PERK and IRE-I in Marc-145 cells by using siRNA. Next, we investigated the consequence of PERK and IRE-I knockdown on autophagy induction. As shown in Fig. 6D,E, PERK knockdown decreased the expression of beclin 1 and LC3-II, and reduced the level of GFP-LC3 accumulation; however, this phenomenon was not observed in siIRE1-treated cells.

### PRRSV infection induces CHOP expression that leads to ER stress-mediated apoptosis

**Figure 7:**
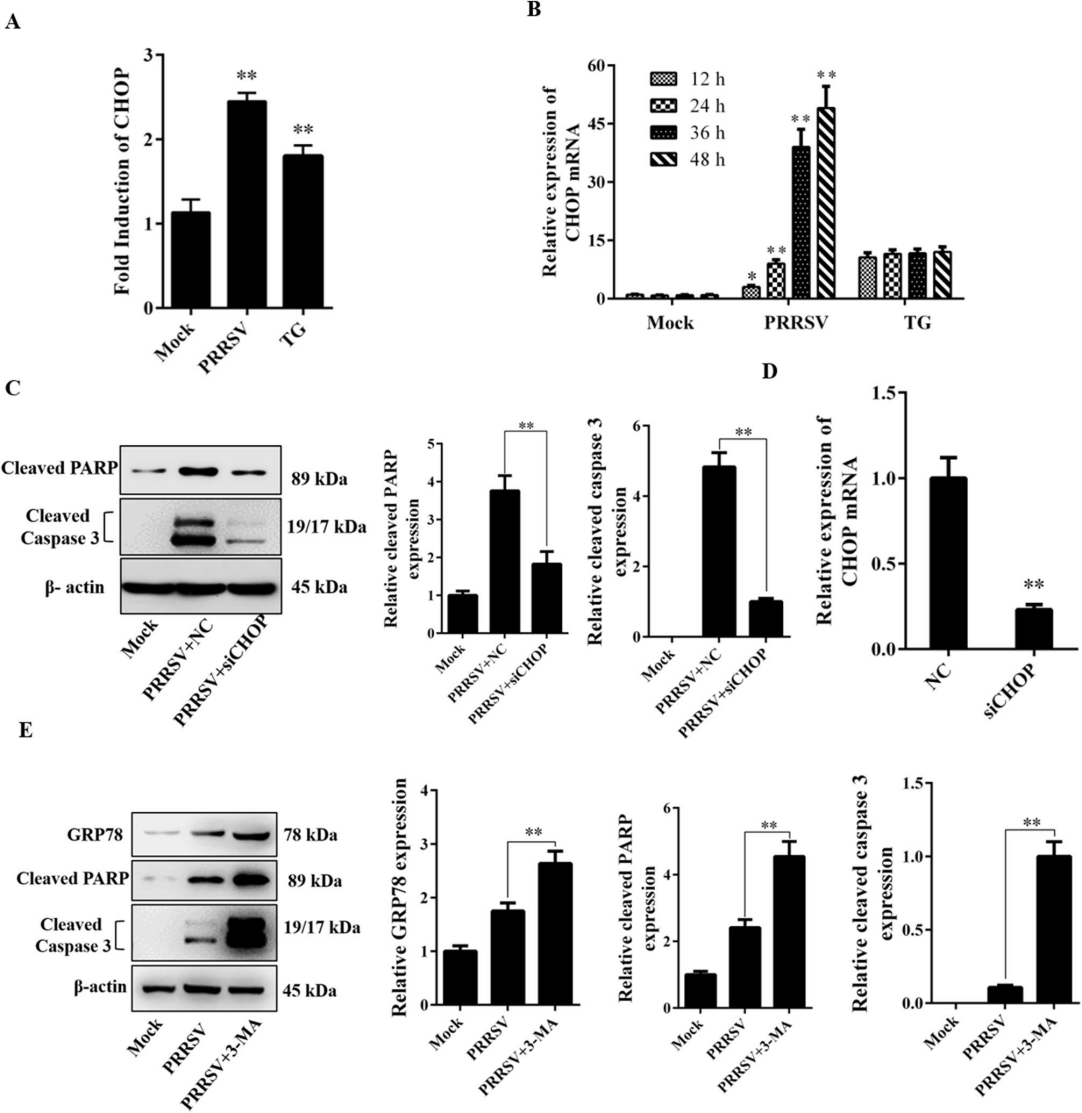
PRRSV infection induces CHOP expression and elicits ER stress-mediated apoptosis. (**A**) Marc-145 cells were transfected with the reporter plasmid CHOP-luc, along with the pRL-TK plasmid, followed by PRRSV infection at an MOI of 0.5. At 36 h after infection, the cells were collected, and luciferase activities were determined. (**B**) Cells were infected with PRRSV, and collected at the indicated time points. Mock-infected and TG (5 μM)-treated cells were considered as negative and positive controls, respectively. The mRNA expression levels of CHOP were evaluated by qRT-PCR. (**C**) Marc-145 cells were transfected with CHOP or NC siRNA for 24 h, and the cells were then infected with PRRSV for 36 h. Cleaved caspase 3 and cleaved PARP level was then measured by western blot assay in whole cell lysates, and the relative expression was analyzed. (**D**) Marc-145 cells were infected with PRRSV for 24 h, and transfected with siRNA. After 24 h, CHOP mRNA levels were assessed by qRT-PCR and normalized to GAPDH expression. (**E**) Marc-145 cells were pretreated with 3-MA for 1 h, and infected with PRRSV for 36 h, the GPR78, cleaved PARP and cleaved caspase 3 expression was detected through western blot. And the relative expression of these proteins was evaluated with ImageJ. Results are expressed as mean ± SD for experiments performed in triplicate. *p < 0.05 and **p < 0.01.

The transcription factor CHOP can be activated in cells experiencing ER stress, and CHOP activation appears to contribute to subsequent cell growth arrest and apoptosis. To investigate the activation of CHOP, Marc-145 cells were cotransfected with CHOP-luc and pRL-TK. At 12 h post-transfection, the cells were further infected or mock-infected with PRRSV, followed by the dual-luciferase assay. As shown in Fig. 7A, the CHOP promoter was activated during PRRSV infection, and the induction was even higher than that observed after treatment with TG for 4 h. Furthermore, we performed qRT-PCR to demonstrate the induction of CHOP at the mRNA level. The results showed that treatment with TG induced the transcription of CHOP, and CHOP mRNA induced by PRRSV was observed at 24 h and sustained until 48 h. The results indicated that PRRSV infection activated the expression of CHOP (Fig. 7B).

To determine whether PRRSV infection induces ER stress-mediated apoptosis, the expression of apoptotic executors cleaved caspase 3 and cleaved poly ADP-ribose polymerase (PARP) were measured by the western blot. We found that caspase 3 and PRAP were activated during PRRSV infection, however, knockdown the expression of CHOP considerably inhibited caspase 3 and PARP activation (Fig. 7C). Under normal conditions, the expression of CHOP mRNA was too low to detect siRNA silencing efficiency. To confirm the silencing efficiency, siRNAs were transfected to the TG-treated cells, and qRT-PCR was performed to detect the silencing efficiency. Compared with the control siRNA, the siRNA targeting CHOP significantly suppressed mRNA expression (Fig. 7D). We next sought to further study the relevance among PRRSV induced autophagy, ER stress, and apoptosis. 3-MA was used to inhibit autophagy during PRRSV infection, and we found that when autophagy was inhibited, PRRSV will induce more GPR78, cleaved caspase 3 and cleaved PARP expression (Fig. 7E), indicating that autophagy was activated to alleviate the ER stress during PRRSV infection.

## Discussion

As the locus for modification and folding of cellular proteins, ER is also an essential organelle for viral replication and maturation. A large number of viral proteins synthesized in virus-infected cells usually leads to the accumulation of proteins and causes ER stress. Several viruses have been shown to induce ER stress and activate the UPR signaling pathways. For instance, West Nile virus and dengue virus activate ER stress and trigger UPR^16,17^. However, many viruses apparently activate a subset or only one of the UPR pathways, while some viruses activate one UPR pathway but suppress others. For example, Japanese encephalitis virus (JEV) and severe acute respiratory syndrome virus can activate IRE1 pathway^18,19^, both IRE1 and ATF6 pathways are induced during rotavirus infection^20^, whereas hepatitis C virus (HCV) suppresses the IRE1-XBP1 pathway even though it activates the PERK- and ATF6-initiated pathways^21,22^. Zuckermann et al. revealed that PRRSV infection can induce phosphorylation of PERK and IRE1,they speculated that PRRSV can induce ER stress and activate UPR^13^. However, they did not clarify whether the ATF6 pathway was activated during PRRSV infection. Furthermore, it remained to be investigated whether the downstream genes of UPR were activated during PRRSV infection and whether UPR activation affects PRRSV replication.

In the present study, by observing the morphology of ER and detecting the expression of ER chaperones, we found that PRRSV infection could induce ER stress and that the stress sensor PERK was phosphorylated. Furthermore, by evaluating the levels of phosphorylated eIF2α and ATF4 mRNA, we confirmed that the PERK signaling pathway was activated during PRRSV infection. Phosphorylated eIF2α leads to repression of protein synthesis. To enhance their protein synthesis, many viruses have evolved distinct mechanisms to counteract eIF2α phosphorylation. For instance, African swine fever virus encodes the DP71L protein and herpes simplex virus encodes the γ134.5 protein; these proteins are highly homologous to GADD34 and have been shown to dephosphorylate eIF2α and block translation shutoff during viral infection^23,24^. Dengue virus infection activates the expression of GADD34 to compensate for the induction of eIF2α phosphorylation. In the present study, we also observed the activation of ATF4,this led us to hypothesize that PRRSV compensates for eIF2α phosphorylation by enhancing the expression of GADD34. To corroborate our hypothesis, we performed qRT-PCR analyses of GADD34 mRNA expression levels, which suggested that PRRSV infection induced the expression of GADD34 at approximately the same time as eIF2α was phosphorylated. We concluded that the upregulation of GADD34 counteracted eIF2α phosphorylation and relieved translation inhibition to facilitate the synthesis of viral proteins.

Some viruses also regulate the IRE1 pathway during their infection. For example, the expression of HCV subgenomic replicons in human hepatoma cells resulted in the activation of IRE1 and led to elevated accumulation and expression of Xbp1s^22^, however, the trans-activating activity of Xbp1s was inhibited. In the present study, we demonstrated that PRRSV replication led to an increase in Xbp1 mRNA splicing. Furthermore, the activation of UPRE and EDEM suggested that the IRE1/Xbp1 pathway was activated during PRRSV infection.

Similar to IRE1, the activation of ATF6 caused transcriptional upregulation of ER chaperone proteins to relieve ER stress. Previous studies suggested that virus infection results in a selective induction of ATF6-regulated UPR pathway. Because the expression of GRP78 and GRP94 can be activated by either ATF6 or Xbp1, we investigated whether the ATF6 pathway was involved in the activation of GRP78 and GRP94 during PRRSV infection. Similar to human cytomegalovirus infection, in our study, we did not find the active form of ATF6 during PRRSV infection. Furthermore, the downstream genes such as calnexin, calreticulin, and ER57 were not induced. We concluded that infection with PRRSV induced ER stress, but not the ATF6 pathway. Some other mechanisms might be involved in the repression of ATF6 activation. A probable explanation for this repression was that the activation of the ATF6 signaling pathway could further increase the production of chaperones that could be harmful to PRRSV replication, such as ERAD through crosstalk of the UPR pathways.

UPR induction usually regulates virus replication. For instance, Baltzis et al. reported that vesicular stomatitis virus (VSV) infection activated the PERK pathway, and the higher replication capacity of VSV in PERK−/− cells resulted from their inability to attenuate viral protein synthesis due to an impaired eIF2α phosphorylation^25^. Here, we demonstrated that the inhibition of UPR by 4-PBA significantly reduced PRRSV yield. The RNAi assay revealed that both PERK and IRE1 played beneficial roles in PRRSV replication. This finding is consistent with the previous report that PRRSV replication was inhibited by the suppression of IRE1 activation^12^. In this study, we did not investigate the specific mechanism by which the UPR enhanced PRRSV replication,we predicted that the activation of PERK pathway-induced apoptosis was a mechanism to ensure subsequent virus exit from the cells and facilitate its replication. We demonstrated that PRRSV induced the activation of GRP78 and GRP94 via the IRE1/Xbp1 pathway, maybe downregulated the IRE1 expression reduced the protein folding capacity, leading inhibition of viral replication.

The transcription program induced by the UPR encompasses additional cellular programs to restore cell damage, such as the induction of autophagy. With regard to PRRSV, several reports, including our report, have demonstrated that autophagy induction was involved during PRRSV infection and that the induction of cellular autophagy can enhance PRRSV replication^26^. In the present study, we found that ER stress inhibitor or siRNA treatment inhibited UPR, which could alleviate PRRSV-activated autophagy. This effect was regulated only by the PERK signaling branch, and siIRE1 treatment did not attenuate PRRSV-induced autophagy. This finding is consistent with the report of Luhr et al. that the UPR affects the autophagic activity via PERK but not through the IRE1 signaling branch^27^. Intriguingly, PRRSV-induced UPR also plays a positive role in its replication, as autophagy can enhance PRRSV replication,thus, the suppression of UPR could possibly decrease PRRSV replication at least partially through the suppression of the autophagy response.

We know that the excess level of UPR will lead to apoptosis. Several studies have revealed that PRRSV infection triggers apoptosis in infected cells^28^. In the present study, we discussed whether ER stress-mediated apoptosis was involved during PRRSV infection. Activated CHOP is a key marker of ER stress-mediated apoptosis^29^, and ER stress-mediated apoptotic signal ultimately converge on caspase 3. Therefore, in this study, we analyzed the activation of CHOP and caspase 3. We were unable to detect cleaved ATF6 during PRRSV infection, but we observed a significant upregulation of CHOP. Because ATF4 and ATF6 work synergistically to activate CHOP expression^30^, we speculated that CHOP upregulation during PRRSV infection may be modulated entirely by ATF4. From the RNAi experiment, we found that the deletion of CHOP resulted in lower activation of caspase 3 during PRRSV infection. On the basis of these findings, we can conclude the crucial role of ER stress in PRRSV-induced apoptosis. Notably, different to apoptosis, autophagy is a protective mechanism for cells, however, both of them were activated during PRRSV infection. We infer that autophagy was activated to eliminate the ER stress in the earlier stage of PRRSV infection, however, the unremitting ER stress leads to cell apoptosis at last.

## Materials and methods

### Cells, viruses, and chemicals

Marc-145 cells were cultured in DMEM (GIBCO) supplemented with 10% heated-inactivated fetal bovine serum (FBS), 100 U/mL penicillin, and 100 μg/mL streptomycin sulfate at 37 °C in 5% CO_2_ incubator. PRRSV strain WUH3 (GenBank accession no. HM853673)^31^, a highly pathogenic type 2 PRRSV that was isolated in China in 2006, was propagated in Marc-145 cells and stored at − 80 °C. The chemicals thapsigargin (TG) and tunicamycin (TU) as the ER stress inducers, 3-MA as the autophagy inhibitor, and 4-phenylbutyric acid (4-PBA) as the ER stress inhibitor were purchased from Sigma-Aldrich (St Louis, MO).

### Plasmids, transfection, and reporter gene assays

The luciferase reporter plasmid Xbp1-luc was constructed by fusion expression of Xbp1 and firefly luciferase. CHOP-luc was under the control of the CHOP promoter. UPRE-luc and ERSE-luc carried the UPR element or the ER stress response element. All the reporter plasmids were gifted by Dr. Zhongbin Chen at Beijing Institute of Radiation Medicine, China. Before transfection, Marc-145 cells were cultured in 24-well plates. When the cells reached approximately 70–80% confluence, the cells were cotransfected with reporter plasmid and pRL-TK plasmid (Promega) for normalization. Twenty-four hours after transfection, the cells were infected with PRRSV for 36 h. Whole-cell lysates were prepared and firefly and Renilla luciferase activities were determined using a dual-luciferase reporter assay system (Promega) according to the manufacturer’s instructions. Data represent relative firefly luciferase activity normalized to Renilla reniformis luciferase activity.

### Immunofluorescence assay

Marc-145 cells were seeded on cover glasses (NEST Biotechnology) in 24 well plates and infected with PRRSV for 24 h. And then, cells were fixed with 4% paraformaldehyde for 10–15 min. After three washes with PBS, the cells were permeabilized with 0.2% Triton X-100 for 5 min, and blocked with 0.5% BSA for 2 h. The cells were subsequently incubated with antibody against calnexin overnight at 4 ℃. After three times washing, the cells were incubated with Alexa Fluor 488 conjugated secondary antibody for 1 h at 37 ℃, and the nuclei staining with DAPI for 10 min. After three times PBS wash, the cells were subjected to image analysis by confocal microscopy.

### Western blotting

Marc-145 cells were infected with PRRSV strain WUH3 at an MOI of 0.5. At 12, 24, 36, and 48 h post-infection (hpi), the cells were washed with cold phosphate-buffered saline (PBS) before the addition of an appropriate volume of lysis buffer. After incubation for 10 min on ice, lysates were harvested into 5× sodium dodecyl sulfate–polyacrylamide gel electrophoresis (SDS-PAGE) sample buffer. Equal amounts of proteins from cell lysates were resolved on 12% SDS-PAGE and transferred to polyvinylidene difluoride membranes (Millipore, Bedford, MA, USA). Densitometry analysis was performed with ImageJ software, and the band intensities were normalized to those of β-actin. Antibodies specific for Calnexin, beclin 1, LC3, GRP78, GRP94, PERK, IRE1, cleaved caspase 3, cleaved PARP, eIF2α, and Phospho-eIF2α were purchased from Cell Signaling Technology (CST). The anti-ATF6 antibody that can recognize both 90- and 50-kDa ATF6 was obtained from Abcam. Monoclonal antibody against the nonstructural protein 2 (Nsp2) of PRRSV was described previously^32^. Anti-actin antibody and all the horseradish peroxidase (HRP)-conjugated secondary antibodies were purchased from Beyotime (China).

### siRNAs, RNA extraction, and qRT-PCR

The siRNA targeting CHOP was purchased from CST, Cells were transfected with siRNA using Lipofectamine 2000 (Invitrogen). Total RNAs were extracted from the cells by using TRIzol reagent and reverse transcribed to cDNA. Quantitative real-time PCR was performed in a LightCycler 480 (Roche) using SYBR Green I Master (Roche), the sequences for primer and siRNA are listed in Supplementary Table S1.

### Plaque assay

Marc-145 cells were cultured in 6-well plates. When cells were grown to 100% confluent, they were infected for 1 h with tenfold serial dilutions of PRRSV-containing samples. After washes with PBS, the cells were overlaid with DMEM (without neutral red) containing 0.9% agarose, and incubated until plaques were visible, about 3–4 days later. The cells were stained with 1% crystal violet and the plaque number was calculated. Virus titers were presented as plaque forming units (PFU)/mL.

### Transmission electron microscopy

For ultrastructural analysis, Marc-145 cells were sham infected or infected with PRRSV at MOI of 0.5. After 36 h, the cells were fixed in 2.5% glutaraldehyde for 20 min. After three times washing in PBS, the cells were post-fixed in 2% osmium tetroxide in 0.1 M sodium cacodylate. The cells were then dehydrated in a graded series of acetone washes and embedded in Agar 100 epoxy resin. Ultrathin sections were cut and viewed on transmission electron microscope (Tecnai G2 Spirit Twin).

### Statistical analysis

All experiments were conducted in triplicate to ensure that the results were reproducible. Data are presented as mean ± standard deviation (SD). A p value of < 0.05 was considered significant, and p < 0.01 was considered highly significant.

## Supplementary information

Supplementary Table S1.
