# Supplementary material for: Porcine reproductive and respiratory syndrome virus infection induces endoplasmic reticulum stress, facilitates virus replication, and contributes to autophagy and apoptosis
Source: Sci Rep. 2020 Aug 4;10:13131. doi: 10.1038/s41598-020-69959-z (PMC7403369; doi:10.1038/s41598-020-69959-z)
Supplement: Supplementary file 1 — Supplementary Table S1. [file 41598_2020_69959_MOESM1_ESM.docx]

**Porcine reproductive and respiratory syndrome virus infection induces endoplasmic reticulum stress, facilitates virus replication, and contributes to autophagy and apoptosis**

**Quangang Chen^1^, Yanjuan Men^1^, Dang Wang^2,3^, Deqin Xu^4^, Suyan Liu^2,3^, Shaobo Xiao^2,3^, Liurong Fang^2,3,^***

^1^ Laboratory Animal Center, Xuzhou Medical University, Xuzhou, 221004, China

^2^ State Key Laboratory of Agricultural Microbiology, College of Veterinary Medicine, Huazhong Agricultural University, Wuhan, 430070, China

^3^ Key Laboratory of Preventive Veterinary Medicine in Hubei Province, the Cooperative Innovation Center for Sustainable Pig Production, Wuhan, 430070, China

^4^ Department of Oncology, The Affiliated Hospital of Xuzhou Medical University, Xuzhou, 221004, China

Corresponding author: Liurong Fang

E-mail: [fanglr@mail.hzau.edu.cn](mailto:fanglr@mail.hzau.edu.cn)

**Table S1. Primers used in this study**

| **Primer** | **Sequence 5'→3'** |
| --- | --- |
| ATF4-F | AGGAGTTCGCCTTGGATGCCCTG |
| ATF4-R | AGTGATATCCACTTCACTGCCCAG |
| GADD34-F | GGAGGAAGAGAATCAAGCCA |
| GADD34-R | TGGGGTCGGAGCCTGAAGAT |
| Xbp1-F | AAACAGAGTAGCAGCTCAGACTGC |
| Xbp1-R | TCCTTCTGGGTAGACCTCTGGGAG |
| EDEM-F | TTGACTCTTGTTGATGCATTGGA |
| EDEM-R | GCTTTCTGGAACTCGGATGAAT |
| ER57-F | CTGTAAGAACCTGGAGCCCAAGT |
| ER57-R | TCATTGGCTGTGGCATCCAT |
| Clan-F | TCCTTGAAGCAAATGTGTGG |
| Clan-R | ACTGTCAACGGAGGGTGAAG |
| Clar-F | CGAGGACTGGGATGAAGAGA |
| Clar-R | AATCTGGGTTGTCGATCTGC |
| PERK-F | TGGCAACCATTGTGCAAATAA |
| PERK-R | AGTGGTTGGTCTTGGAGGAGAA |
| IRE1-F | GACCGTGAGGTCCAGCTGTT |
| IRE1-R | CGATGGCAATGTACTGGAATTG |
| CHOP-F | AGCTGGAACCTGAGGAGAGA |
| CHOP-R | TGGATCAGTCTGGAAAAGCA |
| GAPDH-F | TCATGACCACAGTCCATGCC |
| GAPDH-R | GGATGACCTTGCCCACAGCC |
